# Relation Between Serum Total Cholesterol Level and Cardiovascular Disease Stratified by Sex and Age Group: A Pooled Analysis of 65 594 Individuals From 10 Cohort Studies in Japan

**DOI:** 10.1161/JAHA.112.001974

**Published:** 2012-10-25

**Authors:** Sin-ya Nagasawa, Tomonori Okamura, Hiroyasu Iso, Akiko Tamakoshi, Michiko Yamada, Makoto Watanabe, Yoshitaka Murakami, Katsuyuki Miura, Hirotsugu Ueshima, for the Evidence for Cardiovascular Prevention from Observational Cohorts in Japan (EPOCH-JAPAN) Research Group

**Affiliations:** Department of Epidemiology and Public Health, Kanazawa Medical University, Ishikawa, Japan (S.N.); Department of Preventive Medicine and Public Health, Keio University, Tokyo, Japan (T.O.); Department of Social and Environmental Medicine, Osaka University Graduate School of Medicine, Osaka, Japan (H.I.); Department of Public Health, Aichi Medical University School of Medicine, Aichi, Japan (A.T.); Department of Clinical Studies, Radiation Effects Research Foundation, Hiroshima, Japan (M.Y.); Department of Preventive Cardiology, National Cerebral and Cardiovascular Center, Suita, Japan (M.W.); Department of Medical Statistics, Shiga University of Medical Science, Otsu, Japan (Y.M.); Department of Health Science, Shiga University of Medical Science, Otsu, Japan (K.M.); Life-Related Disease Prevention Center, Shiga University of Medical Science, Otsu, Japan (H.U.)

**Keywords:** cholesterol, coronary heart disease, pooled analysis, stroke

## Abstract

**Background:**

The relation between serum total cholesterol (TC) and cardiovascular disease in women and in the elderly is unclear, especially in Asian populations.

**Methods and Results:**

We examined this relation in the largest-scale pooled analysis of the Japanese population, the Evidence for Cardiovascular Prevention from Observational Cohorts in Japan (EPOCH-JAPAN) study. A total of 65 594 participants who were 40 to 89 years of age and did not have a past history of cardiovascular disease were examined. Cox proportional-hazards models were used to estimate hazard ratios for death from total stroke, cerebral infarction, intracranial cerebral hemorrhage, or coronary heart disease. The mean follow-up period was 10.1 years, with the number of deaths from total stroke, cerebral infarction, cerebral hemorrhage, and coronary heart disease being 875, 457, 212, and 374, respectively. The participants were divided into 2 age groups: middle-aged (40 to 69 years; mean age 55 years) and elderly (70 to 89 years; mean age 75 years). In men, the multivariate-adjusted hazard ratios for coronary heart disease in the highest TC category (≥6.21 mmol/L) compared with the lowest category (<4.14 mmol/L) were 2.52 (95% confidence interval: 1.15–5.07) in middle-aged participants and 2.77 (1.09–7.03) in elderly participants. In women, the hazard ratios of the highest TC category (≥6.72 mmol/L) compared with the lowest category (<4.66 mmol/L) were 3.20 (1.44–7.09) in middle-aged participants and 1.02 (0.42–2.49) in elderly participants. TC levels were not associated with cerebral infarction in any age or sex group and were associated negatively with total stroke and cerebral hemorrhage.

**Conclusion:**

High serum TC levels are associated with coronary heart disease in middle-aged Japanese men and women, but evidence in elderly Japanese individuals is still limited.

## Introduction

Hypercholesterolemia is a well-documented and established risk factor for coronary heart disease (CHD).^1,2^ However, evidence of this association is mainly from middle-aged or relatively young elderly men <70 years of age, whereas evidence from women or the elderly, especially in Asian populations, is scarce. One large meta-analysis based on observational studies found that high levels of serum total cholesterol (TC) were associated with an increased CHD mortality rate in both men and women.^3^ However, this study was stratified only by age and sex and was not adjusted for other confounders. Another meta-analysis based on observational studies showed a weaker association between TC and CHD in women and in participants ≥75 years of age.^4^ To our knowledge, no observational study has demonstrated a clear positive relation between serum TC levels and death from CHD in women and the elderly specific to Asian populations.^1^ Furthermore, less evidence is available on the influence of serum TC on stroke than on CHD.

We therefore investigated the associations between serum TC level and death due to cardiovascular disease (CVD), such as CHD and stroke, after stratification by sex and age in the largest-scale pooled analysis carried out in the Japanese population. Our a priori hypothesis is that a high serum TC level is a risk factor for CVD in Japanese after stratification by both sex and age.

## Methods

### Study Design

This study was part of a pooled project called EPOCH-JAPAN (Evidence for Cardiovascular Prevention from Observational Cohorts in Japan), which incorporates a meta-analysis of individual participant data from 13 cohorts across Japan. The project was designed to conduct pooled analyses and examine the relation between cause-specific mortality rate and various exposures, including laboratory measures and lifestyle factors. The guidelines for a cohort recruitment of EPOCH-JAPAN were as follows: collection of health examination measures, >1000 participants, and >10 years of follow-up (although each cohort's collaborator had discretion to choose the size of his or her data set for pooling). Both nationwide and regional cohort studies were included. We have reported the detailed characteristics of each cohort previously.^5^

### Study Population

Of the 13 cohorts, 10 provided data on the cause of death (n=90 528).^5^ The people who were (1) <40 or ≥90 years of age (n=10 528), (2) had past history of CVD (n=7422), and (3) lacked data on TC level (n=2122) at the baseline survey were removed. Moreover, 4862 participants were removed because of missing data for at least one of the following covariates: sex, age, body mass index, blood pressure, and smoking and drinking status. Finally, a total of 65 594 participants were included in the analysis. The levels of serum TC were measured enzymatically in all the cohorts, with the exception of the NIPPON DATA80 cohort, in which TC was measured by the Lieberman-Burchard direct method.

### Ascertainment of Death

The causes of death were sought in great detail from the available sources in each cohort study. In most studies, death certificates were reviewed or the National Vital Statistics were used after permission had been obtained. Other sources used in some studies included autopsy reports, medical records, health examinations, and questionnaires. The underlying cause of death was coded according to the *International Classification of Diseases* (*ICD*) for National Vital Statistics based on the criteria proposed by the World Health Organization.^6^ These classifications were based on the *ICD-9* until the end of 1994 and on the *ICD-10* from the beginning of 1995. The respective classification codes for *ICD-9* and *ICD-10* used in the study were as follows: death from CVD (390 to 459; I00 to I99), total stroke (TS) (410 to 414 or 430 to 438; I20 to I25 or I60 to I69), cerebral infarction (433 or 434 or 437.8; I63 or I69.3), intracranial cerebral hemorrhage (431 to 432; I61 or I69.1), and CHD (410 to 414; I20 to I25).

### Statistical Methods

Sex-specific analysis was performed. TC was categorized into 7 categories (<4.14, 4.14 to 4.65, 4.66 to 5.16, 5.17 to 5.68, 5.69 to 6.20, 6.21 to 6.71, and ≥6.72 mmol/L) in accordance with a previous Japanese cohort study,^7^ which had provided key evidence for the guidelines of the Japan Atherosclerosis Society for diagnosis and prevention of atherosclerotic CVD for Japanese.^8^ However, because only a small number of participants had TC levels ≥6.72 mmol/L in men and <4.14 mmol/L in women, with the number of events in these participants being limited, we decided to combine these TC levels into the adjacent category (6.21 to 6.71 mmol/L in men and 4.14 to 4.65 mmol/L in women). The lowest level in both sexes (men, <4.14 mmol/L; women, <4.65 mmol/L) served as the reference group.

The study population was divided into 2 age groups in both men and women: middle-aged (40 to 69 years; mean age 55 years) and elderly (70 to 89 years; mean age 75 years). Age group– and sex-specific analyses were performed. Cox proportional-hazards models stratified by cohorts^9^ were used to estimate the hazard ratios (HRs) for cardiovascular outcomes according to baseline TC. Deaths from CHD and from TS and its subtypes (cerebral infarction and cerebral hemorrhage) were used in the analysis. In the Cox model, age, body mass index, systolic blood pressure, smoking status (current smoker, ex-smoker, never-smoker), and drinking status (current drinker, ex-drinker, never-drinker) were used as confounding variables.

All confidence intervals were estimated at the 95% level, and the significance level was set at *P*=0.05. All the statistical analyses were performed in Statistical Analysis System release 9.13 (SAS Institute, Inc., Cary, NC).

## Results

The baseline characteristics of the participants in the 10 cohorts are shown in Table 1. Each baseline survey was performed between 1977 and 1990, with the number of participants ranging from 1608 in the Tanno-Sobetsu cohort to 24 940 in the Japan Collaborative Cohort (JACC) study. Mean age ranged from 47 years in the YKK cohort to 61 years in the Osaki cohort.

**Table 1.** Baseline Characteristics of the Study Participants in Each Cohort

										Smoking*Status			Drinking† Status		

Cohort Name	Geographic Location (Prefecture)	Year of Baseline Survey	Follow-Up Periods, y, Average±SD	No. Participants	Age at Study Entry, y, Average±SD	Serum Total Cholesterol, mmol/L, Average±SD	Systolic Blood Pressure, mm Hg, Average±SD	Diastolic Blood Pressure, mm Hg, Average±SD	Body Mass Index, kg/m^2^, Average±SD	Never	Ex-	Current	Never	Ex-	Current
**Men**															

Tanno-Sobetsu	Hokkaido	1977	18.5±3.7	742	50.5±6.9	4.81±1.06	131±19	82±10	23.1±2.7	226	0	516	212	0	530

Osaki	Miyagi	1994	6.0±1.4	6142	62.1±10.0	5.02±0.88	132±17	80±11	23.6±2.9	1335	1811	2996	966	473	4703

Ohasama	Iwate	1987	9.9±2.6	877	59.4±10.9	4.84±0.88	134±17	76±11	23.1±2.8	435	0	442	341	0	536

Oyabe	Ishikawa	1988	9.6±2.1	1461	60.4±10.3	4.71±0.85	131±20	79±11	22.6±2.7	661	0	800	374	0	1087

YKK workers	Toyama	1990	10.7±2.8	1970	47.3±5.4	5.22±0.88	121±16	76±12	22.7±2.6	545	303	1122	359	30	1581

RERF cohort	Hiroshima	1986	15.4±3.5	619	54.5±10.8	5.12±0.88	122±13	80±8	21.7±2.7	95	182	342	112	35	472

Hisayama	Fukuoka	1988	10.7±2.8	1106	58.3±11.6	5.10±1.06	135±20	81±11	22.8±3.0	226	327	553	369	69	668

JACC study	Nationwide‡	1988–1990	9.4±2.1	8988	57.9±9.9	4.86±0.91	135±19	81±11	22.8±2.8	2048	2163	4777	1760	436	6792

NIPPON DATA80	Nationwide‡	1980	16.5±4.7	2737	55.5±10.7	4.84±0.88	142±21	85±12	22.5±2.9	492	556	1689	556	164	2017

NIPPON DATA90	Nationwide‡	1990	9.4±1.9	2412	57.0±11.3	5.15±0.96	140±20	85±12	23.0±3.0	524	597	1291	820	156	1436

Total			9.9±4.1	27 054	57.7±10.7	4.95±0.91	134±19	81±12	23.0±2.9	6587	5939	14 528	5869	1363	19 822

**Women**															

Tanno-Sobetsu	Hokkaido	1977	18.7±3.6	866	50.3±6.7	5.02±0.91	133±20	82±10	24.2±3.4	801	0	65	790	0	76

Osaki	Miyagi	1994	6.0±1.5	6612	61.2±9.2	5.48±0.88	130±18	78±11	24.1±3.2	6195	106	311	5049	183	1380

Ohasama	Iwate	1987	10.7±2.2	1363	58.4±9.3	5.30±0.93	129±16	73±11	23.9±3.3	1329	0	34	1280	0	83

Oyabe	Ishikawa	1988	10.1±1.4	3166	58.0±9.5	5.22±0.93	126±20	75±11	23.2±3.1	3085	0	81	2729	0	437

YKK workers	Toyama	1990	11.0±2.6	1036	47.2±5.4	5.30±0.96	117±16	72±12	22.3±2.7	1025	2	9	825	2	209

RERF cohort	Hiroshima	1986	16.2±2.6	1342	57.3±9.9	5.56±0.98	121±14	76±9	22.3±3.3	1168	35	139	803	17	522

Hisayama	Fukuoka	1988	11.3±2.2	1513	59.4±11.8	5.53±1.06	133±22	76±11	22.9±3.3	1378	31	104	1363	17	133

JACC study	Nationwide‡	1988–1990	9.6±1.9	15 952	56.5±9.5	5.25±0.93	132±19	78±11	23.3±3.2	15 213	182	557	12 800	155	2997

NIPPON DATA80	Nationwide‡	1980	17.3±4.0	3415	55.8±10.7	5.04±0.88	138±22	81±12	23.1±3.4	3052	73	290	2753	42	620

NIPPON DATA90	Nationwide‡	1990	9.6±1.5	3275	56.8±11.5	5.48±0.98	137±20	81±12	23.1±3.3	2925	72	278	3055	26	194

Total			10.3±3.9	38 540	57.2±10.1	5.33±0.96	131±20	78±11	23.4±3.2	36 171	501	1868	31 447	442	6651

* In the studies of Tanno-Sobetsu, Ohasama, and Oyabe, ex-smokers were classified as nonsmokers.

† In the studies of Tanno-Sobetsu, Ohasama, and Oyabe, ex-drinkers were classified as nondrinkers.

‡ In this nationwide cohort study, the participants were from all areas of Japan.

The number of total participants was 65 594 (27 054 men and 38 540 women), and the mean age was 57 years. The mean ± standard deviation (SD) serum TC level of the total participants was 4.95±0.91 mmol/L for men and 5.33±0.96 mmol/L for women. The levels were lowest in the Oyabe cohort for men (4.71 mmol/L) and in the Tanno-Sobetsu cohort for women (5.02 mmol/L) and were highest in the YKK cohort for men (5.22 mmol/L) and in the Radiation Effects Research Foundation (RERF) cohort for women (5.56 mmol/L). The mean follow-up period was ≍10.1 years, with the number of deaths from TS, cerebral infarction, cerebral hemorrhage, and CHD being 875, 457, 212, and 374, respectively.

In the Cox regression models, the relation between serum TC levels and CHD death was continuous and positive overall, with the exception of elderly women. However, when TC level was treated as a continuous variable, the relation was not significant in elderly men (Table 2). In middle-aged men, the multivariate-adjusted HR of the highest TC category (≥6.21 mmol/L) for CHD was 2.52 (95% confidence interval [CI]: 1.15–5.07) compared with the lowest TC category (<4.14 mmol/L), and the multivariate-adjusted HR for a 1-SD increment in serum TC level (0.98 mmol/L) was 1.26 (95% CI: 1.11–1.42). In elderly men, the multivariate-adjusted HR of the highest TC category for CHD was 2.77 (95% CI: 1.09–7.03) compared with the lowest TC category. The multivariate-adjusted HR for a 1-SD increment in serum TC level in these participants was 1.23 (95% CI: 0.96–1.56).

**Table 2.** The Number of Deaths, Crude Mortality Rate, and Multivariate-Adjusted HRs for CVD Death According to Total Cholesterol Levels

	Age Category, years		Total Cholesterol, mmo/L							
			
			<4.14	4.14 to 4.65	4.66 to 5.16	5.17 to 5.68	5.69 to 6.20	6.21 to 6.71	≤6.72	For 1 SD* Increasing
**Men**										

Coronary heart disease	40 to 69	Number of participants	3956	4805	5339	4276	2597	2111		
		
		Number of deaths	16	30	24	25	19	17		
		
		Crude mortality rate†	0.39	0.60	0.44	0.57	0.72	0.82		
		
		HR (95% CI)‡	1	1.56 (0.85 to 2.87)	1.20 (0.64 to 2.28)	1.71 (0.91 to 3.24)	2.26 (1.14 to 4.45)	2.52 (1.15 to 5.07)		1.26 (1.11 to 1.42)

	70 to 89	Number of participants	871	860	892	694	380	273		
		
		Number of deaths	11	21	18	11	10	8		
		
		Crude mortality rate†	1.70	3.18	2.69	2.11	3.45	5.08		
		
		HR (95% CI)‡	1	1.95 (0.93 to 4.06)	1.73 (0.81 to 3.67)	1.49 (0.64 to 3.48)	2.31 (0.96 to 5.53)	2.77 (1.09 to 7.03)		1.23 (0.96 to 1.56)

Cerebral infarction	40 to 69	Number of participants	3956	4805	5339	4276	2597	2111		
		
		Number of deaths	16	34	25	14	9	7		
		
		Crude mortality rate†	0.39	0.67	0.45	0.32	0.34	0.32		
		
		HR (95% CI)‡	1	1.78 (0.98 to 3.23)	1.26 (0.67 to 2.37)	1.05 (0.51 to 2.16)	1.13 (0.49 to 2.59)	1.11 (0.45 to 2.73)		0.92 (0.74 to 1.14)

	70 to 89	Number of participants	871	860	892	694	380	273		
		
		Number of deaths	37	32	43	28	14	8		
		
		Crude mortality rate†	5.73	4.85	6.43	5.38	4.82	6.47		
		
		HR (95% CI)‡	1	0.85 (0.53 to 1.38)	1.25 (0.80 to 1.96)	1.25 (0.76 to 2.07)	0.94 (0.50 to 1.77)	0.78 (0.36 to 1.69)		1.04 (0.87 to 1.24)

**Women**										

Coronary heart disease	40 to 69	Number of participants	8566		7230	7240	5359	3159	2502	
		
		Number of deaths	12		16	15	15	8	15	
		
		Crude mortality rate†	0.13		0.21	0.20	0.28	0.25	0.59	
		
		HR (95% CI)‡	1		1.37 (0.65 to 2.90)	1.21 (0.56 to 2.61)	1.56 (0.72 to 3.36)	1.45 (0.58 to 3.59)	3.20 (1.44 to 7.09)	1.36 (1.12 to 1.66)

	70 to 89	Number of participants	945		884	968	789	482	416	
		
		Number of deaths	20		19	17	11	9	7	
		
		Crude mortality rate†	2.37		2.46	2.02	1.64	2.15	1.92	
		
		HR (95% CI)‡	1		1.08 (0.57 to 2.03)	0.95 (0.49 to 1.82)	0.83 (0.40 to 1.75)	1.06 (0.48 to 2.37)	1.02 (0.42 to 2.49)	1.02 (0.82 to 1.27)

Cerebral infarction	40 to 69	Number of participants	8566		7230	7240	5359	3159	2502	
		
		Number of deaths	13		13	9	14	8	8	
		
		Crude mortality rate†	0.14		0.17	0.12	0.26	0.25	0.32	
		
		HR (95% CI)‡	1		0.97 (0.45 to 2.10)	0.61 (0.26 to 1.44)	1.12 (0.52 to 2.42)	1.11 (0.45 to 2.74)	1.28 (0.51 to 3.22)	1.08 (0.83 to 1.39)

	70 to 89	Number of participants	945		884	968	789	482	416	
		
		Number of deaths	35		21	27	29	6	7	
		
		Crude mortality rate†	4.16		2.72	3.21	4.32	1.43	1.92	
		
		HR (95% CI)‡	1		0.70 (0.41 to 1.21)	0.89 (0.54 to 1.48)	1.39 (0.84 to 2.29)	0.45 (0.19 to 1.07)	0.69 (0.30 to 1.58)	0.97 (0.80 to 1.16)

**Men and Women**										

Total stroke	40 to 89	Number of participants	8449	11 554	14 345	13 178	9125	5100	3843	
		
		Number of deaths	147	183	182	160	103	45	55	
		
		Crude mortality rate†	1.70	1.52	1.25	1.21	1.14	0.89	1.45	
		
		HR (95% CI)‡	1	0.95 (0.76 to 1.18)	0.82 (0.65 to 1.02)	0.83 (0.66 to 1.05)	0.78 (0.60 to 1.02)	0.60 (0.42 to 0.84)	0.99 (0.72 to 1.37)	0.93 (0.862 to 0.997)

Cerebral hemorrhage	40 to 89	Number of participants	8449	11 554	14 345	13 178	9125	5100	3843	
		
		Number of deaths	44	41	47	38	15	12	15	
		
		Crude mortality rate†	0.51	0.34	0.32	0.29	0.17	0.24	0.39	
		
		HR (95% CI)‡	1	0.71 (0.46 to 1.09)	0.69 (0.46 to 1.05)	0.63 (0.41 to 0.99)	0.37 (0.20 to 0.68)	0.51 (0.27 to 0.99)	0.85 (0.46 to 1.58)	0.84 (0.72 to 0.97)

HR indicates hazard ratio; CVD, cardiovascular disease.

* One SD of total cholesterol was 0.98 mmol/L.

† Crude mortality rate was expressed as per 1000 person-years.

‡ HR was adjusted for age, systolic blood pressure, body mass index, smoking categories, and drinking categories. All analyses were stratified by cohort.

In middle-aged women, the multivariate-adjusted HR of the highest TC category (≥6.72 mmol/L) for CHD was 3.20 (95% CI: 1.44–7.09) compared with the lowest TC category (<4.66 mmol/L), whereas the multivariate-adjusted HR for a 1-SD increment in serum TC (0.98 mmol/L) was 1.36 (95% CI: 1.12–1.66). However, in elderly women, the multivariate-adjusted HR of the highest TC level for CHD was 1.02 (95% CI: 0.42–2.49), and the multivariate-adjusted HR for a 1-SD increment in serum TC was 1.02 (95% CI: 0.82–1.27).

**Figure 1.**
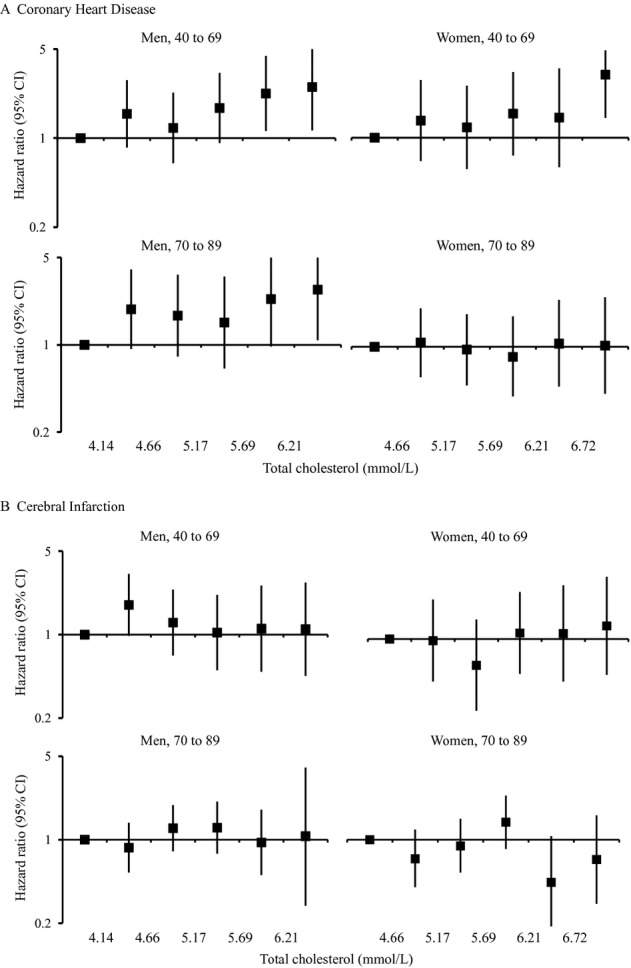
Multivariate-adjusted hazard ratios for death from (A) coronary heart disease and (B) cerebral infarction according to total cholesterol levels. Hazard ratio was adjusted for cohort, age, systolic blood pressure, body mass index, and smoking and drinking categories. CI indicates confidence interval.

On the other hand, serum TC levels were not associated with cerebral infarction in any age or sex group and were associated negatively with cerebral hemorrhage and TS. The multivariate-adjusted HRs for cerebral infarction for a 1-SD increment in serum TC were 0.92 (95% CI: 0.74–1.14) in middle-aged men, 1.04 (95% CI: 0.87–1.24) in elderly men, 1.08 (95% CI: 0.83–1.39) in middle-aged women, and 0.97 (95% CI: 0.80–1.16) in elderly women. The multivariate-adjusted HR for cerebral hemorrhage for a 1-SD increment in serum TC was 0.84 (95% CI: 0.72–0.97) in the combined participants. The multivariate-adjusted HR for TS for a 1-SD increment in serum TC was 0.93 (95% CI: 0.862–0.997) in the combined participants. The Figure 1 summarizes the associations with CHD and cerebral infarction by sex and age groups.

Although 52% (n=34 379) of the participants in the present study had information on antihypertensive medication, the relations between TC and death from CVD (HRs) did not change substantially when use of hypertension medication (16%) was added as a covariate. In the subgroup analysis of participants with information on self-reported diabetes (n=28 793) or casual blood glucose (n=32 384), the relations between TC and death from CVD also were not altered when diabetes was added as a covariate. Because it is likely that there were time period differences in CVD event rates, we reanalyzed the data excluding 2 cohorts with earlier baseline surveys (Tanno-Sobetsu [1977] and NIPPON DATA80 [1980] cohorts). This analysis showed that the relations between TC and death from CVD (HRs) did not change substantially (data not shown).

## Discussion

In this large cohort study in Japan, we found a positive relation between serum TC level and CHD death in both middle-aged women and middle-aged men. We also observed that the highest TC group (≥6.21 mmol/L) had an increased risk for CHD in elderly men, although the multivariate-adjusted HR did not reach a statistically significant level when TC level was treated as a continuous variable. There was also no relation between TC and CHD in elderly women. Except for elderly women, our results are similar to those reported in 2 previous large-scale studies.^3,4^ To our knowledge, this is the first finding from a large-scale study specific to an Asian population that demonstrates a positive relation between hypercholesterolemia and CHD in middle-aged women with a mean age of 55 years. On the other hand, serum TC levels were not associated with cerebral infarction in any age or sex group and were associated negatively with TS and cerebral hemorrhage death.

The Prospective Study of Pravastatin in the Elderly at Risk (PROSPER) study, which included participants between 70 and 82 years of age with prior vascular disease (mean age 75 years), showed that pravastatin reduced the risk of CHD events.^10^ In addition, the Management of Elevated Cholesterol in the Primary Prevention Group of Adult Japanese (MEGA) study showed that pravastatin reduced the risk of CHD in Japanese participants ≥60 years of age without a history of CHD or stroke.^11^ The Justification for the Use of Statins in Primary Prevention: An Intervention Trial Evaluating Rosuvastatin (JUPITER) study also showed that rosuvastatin reduced the incidence of major cardiovascular events, including CHD, in people ≥70 years of age without prior vascular disease who had an elevated high-sensitivity C-reactive protein without apparent hyperlipidemia.^12^ However, observational studies on elderly people at low risk or in the primary care setting are very rare, and there is little evidence of a sex difference. Moreover, elderly people, especially elderly women, are the dominant population in the currently aging societies of developed countries. Therefore, the sex-specific findings we observed in elderly community dwellers are important and suggest an increased risk for CHD in the highest TC group (≥6.21 mmol/L) in elderly men. However, this relation was not significant when TC level was treated as a continuous variable.

The Seven Countries Study showed that Japan had the lowest CHD mortality rate among developed countries, which was attributed largely to remarkably low serum TC levels in the 1950s.^13^ However, changes in lifestyle toward a Westernized pattern in Japan have resulted in a continuous increase in dietary fat intake and serum TC levels. Moreover, it was reported recently that Japanese born after World War II had serum TC levels similar to those of white men^14^ and that the incident rate of CHD in Japan is increasing in some areas.^15,16^ In the Western population, a positive relation between TC and CHD was observed in both sexes and in all age groups, although its association was attenuated in the elderly.^3^ The weak association or lack of association between serum TC and CHD in elderly Japanese participants could be due to relatively less exposure to hypercholesterolemia in their young and middle-aged periods before the baseline, a time when mean serum TC level was known to be very low.^17,18^ Although we do not have information on serum TC levels before the baseline measurements in the present study, elderly Japanese, even those with hypercholesterolemia, might not have had higher serum TC levels throughout their entire lives. Especially for elderly women, the exposure period to high serum TC could be shorter than for men, as serum TC levels in women before menopause are considerably lower than in men. This could be one reason for the lack of relation between serum TC levels and CHD in elderly women. Furthermore, our findings could be explained by survivor bias.^19^ In other words, elderly participants in the present study might have some beneficial characteristics that helped them avoid CHD due to hypercholesterolemia.

In the present study, serum TC levels were not associated with cerebral infarction in any age or sex group. This finding is different from several other large-scale cohort studies in Western populations, which showed a weak but positive association between serum TC and cerebral infarction,^3,4,20,21^ but is similar to previous cohort studies in Japan and some Western countries.^2,22–25^ These discrepancies could be due to differences in the prevalence of subtypes of cerebral infarction. Cerebral infarction consists of 3 major pathological subtypes—namely lacunar, atherothrombotic, and cardioembolic infarctions. In some Western populations, atherothrombotic infarctions account for approximately one half of cerebral infarctions,^26^ whereas in some Japanese populations, it accounts for only approximately one quarter of cerebral infarctions. Furthermore, cardioembolic infarction is more common than atherothrombotic infarction, accounting for 23% to 38% of cerebral infarctions.^27–30^ The Hisayama study in a Japanese community showed that serum low-density lipoprotein cholesterol was associated positively with only atherothrombotic infarctions, whereas it was associated negatively with cardioembolic infarction and showed no association with lacunar infarction.^27^ One possible mechanism for the inverse association between low TC and cardioembolic infarction is that low TC increases the occurrence of atrial fibrillation,^31^ which is the predominant risk factor for cardioembolic infarction. The aforementioned heterogeneity in pathological background of cerebral infarction might be a major reason for the variation in findings observed among cohort studies.

In the present study, serum TC levels were associated negatively with risk of cerebral hemorrhage death. This result is similar to previous studies in Japan and the United States.^20,32,33^ Low serum TC can induce angionecrosis, possibly in coexistence with hypertension. Experimental evidence from one study showed that a hypercholesterolemic diet given to spontaneously hypertensive rats reduced angionecrosis of smooth muscle cells in intracerebral arteries, leading to the occurrence of hemorrhagic stroke.^34^ Low serum TC also can reflect nutritional status, which is known to be related to death after onset. Further basic, clinical, and epidemiological studies on these associations are required. Consequently, because TC can be associated inversely with the cardioembolic type of cerebral infarction in addition to cerebral hemorrhage, it is not unexpected that TC was associated inversely with TS death in the present study, similar to another recent report from Japan.^35^

Several limitations need to be considered when these results are interpreted. First, we did not take into account the use or nonuse of cholesterol-lowering therapy, including statins, the main drug used to treat hypercholesterolemia. However, baseline surveys in 7 cohorts of EPOCH-JAPAN were performed before the introduction of the first statin in Japan (1989).^36^ Another 3 cohorts were also started around 1990, and therefore it is likely that only a few participants were taking statins at baseline. Consequently, it is not likely that this limitation would have changed our inferences substantially. Second, the results were based on single health examinations and were likely to have underestimated the true association because of regression dilution bias. Third, the participants in the study volunteered to receive their health examinations, and for that reason their characteristics could be somewhat different from those of nonparticipants or the general population. This would influence the absolute measure of effect (mortality rate) and could therefore underestimate the risk. However, these differences have little effect on relative measures of effect, such as HRs. Fourth, the number of deaths from CHD in the study might not be sufficient in elderly women to estimate the association with serum TC.

In conclusion, this largest-scale pooled analysis specific to Asians found a significant positive relation between serum TC and CHD in both middle-aged men and middle-aged women who were 40 to 69 years of age and did not have a past history of CVD, although a similar relation in elderly participants was not confirmed. Further research is therefore warranted in elderly men and women.
